# Successful cloning of an adult breeding boar from the novel Chinese Guike No. 1 swine specialized strain

**DOI:** 10.1007/s13205-016-0525-4

**Published:** 2016-10-08

**Authors:** Jun-yu Nie, Xiang-xing Zhu, Bing-kun Xie, Su-qun Nong, Qing-yan Ma, Hui-yan Xu, Xiao-gan Yang, Yang-qing Lu, Ke-huan Lu, Yu-ying Liao, Sheng-sheng Lu

**Affiliations:** 1 State Key Laboratory for Conservation and Utilization of Subtropical Agro-Bioresources, Guangxi High Education Key Laboratory for Animal Reproduction and Biotechnology, College of Animal Science and Technology, Guangxi University, Nanning, 530004 China; 2 Guangxi Key Laboratory of Livestock Genetic Improvement, Guangxi Institute of Animal Sciences, Nanning, 530001 China

**Keywords:** Cloning, Adult breeding boar, Chinese Guike No. 1 pig breed, Swine specialized strain, Pig production industry

## Abstract

Somatic cloning, also known as somatic cell nuclear transfer (SCNT), is a promising technology which has been expected to rapidly extend the population of elaborately selected breeding boars with superior production performance. Chinese Guike No. 1 pig breed is a novel swine specialized strain incorporated with the pedigree background of Duroc and Chinese Luchuan pig breeds, thus inherits an excellent production performance. The present study was conducted to establish somatic cloning procedures of adult breeding boars from the Chinese Guike No. 1 specialized strain. Ear skin fibroblasts were first isolated from a three-year-old Chinese Guike No. 1 breeding boar, and following that, used as donor cell to produce nuclear transfer embryos. Such cloned embryos showed full in vitro development and with the blastocyst formation rate of 18.4 % (37/201, three independent replicates). Finally, after transferring of 1187 nuclear transfer derived embryos to four surrogate recipients, six live piglets with normal health and development were produced. The overall cloning efficiency was 0.5 % and the clonal provenance of such SCNT derived piglets was confirmed by DNA microsatellite analysis. All of the cloned piglets were clinically healthy and had a normal weight at 1 month of age. Collectively, the first successful cloning of an adult Chinese Guike No. 1 breeding boar may lay the foundation for future improving the pig production industry.

## Introduction

Somatic cloning, also known as somatic cell nuclear transfer (SCNT), is a promising technology which can be simply summarized as that transferring the nucleus of a somatic cell into an enucleated oocyte to reconstruct a new embryo, which can then be transplanted into a surrogate mother to generate an offspring (Wilmut et al. 2002; Gurdon and Wilmut 2011). In theory, using somatic cells from a live animal, SCNT technique can be used to produce a large number of offspring with exactly the same genetic makeup from the donor animal. Therefore, since the first batch of cloned pigs generated by SCNT in 2000 (Betthauser et al. 2000; Onishi et al. 2000; Polejaeva et al. 2000), such technique has been expected to rapidly extend the population of elaborately selected breeding boars with superior production performance (Vajta and Gjerris 2006; Vajta et al. 2007; Whyte and Prather 2011; Galli et al. 2012; Niemann and Lucas-Hahn 2012; Lee and Prather 2013; Liu et al. 2015).

Chinese Guike No. 1 pig breed is a novel swine specialized strain generated by crossbreeding of Duroc (male parent) with Chinese Luchuan pig breed (female parent). The later is a unique Chinese indigenous swine breed with numerous production advantages, such as higher resistance to disease, strong adaptive capacity to environmental change, as well as has a large litter size (Fang et al. 2004; Yang et al. 2006; Wang et al. 2014). Therefore, the Chinese Guike No. 1 pig breed which is incorporated with the pedigree background of Duroc and Luchuan pig breeds, resulting in an excellent production performance. In consideration of the traditional breeding process is prolonged and money-consuming, it’s expectable that somatic cloning could show its capabilities in improving the production and extension of adult Chinese Guike No. 1 breeding boars.

The present study was conducted to establish somatic cloning procedures of adult Chinese Guike No. 1 breeding boars for improving their application in pig production industry. We first isolated the ear skin fibroblasts from a three-year-old Chinese Guike No. 1 breeding boar. Cultured cells were then used as donor cells to produce cloned embryos. Such cloned embryos showed full in vitro development and with the blastocyst formation rate of 18.4 % (37/201, three independent replicates). After transferring a total of 1187 nuclear transfer derived embryos to four surrogate recipients, two of them became pregnant and gave birth to six live male offspring with normal health and development were produced. The overall cloning efficiency was 0.5 % and the clonal provenance of such piglets was confirmed by DNA microsatellite analysis.

In summary, in the present study, we successfully produced six healthy cloned piglets using the ear fibroblasts isolated from an adult Chinese Guike No. 1 breeding boar, laying the foundation for improving the application of such breeding boars with superior production performance in pig production industry.

## Materials and methods

### Animal ethics

All animal procedures used in this study were carried out in accordance with the *Guide for Care and Use of Laboratory Animals* (8^th^ edition, released by the National Research Council, USA) and were approved by the Institutional Animal Care and Use Committee (IACUC) of Guangxi University. One healthy adult Chinese Guike No. 1 breeding boar (3 years old and earmarked as #200) was used to establish primary ear skin fibroblasts. Pig ovaries for producing in vitro maturated oocytes used as SCNT recipients were collected from a slaughterhouse in the suburban area near Nanning city, China.

### Reagents and chemicals

Unless otherwise stated, all organic and inorganic reagents were purchased from Sigma-Aldrich Co. (St. Louis, MO, USA). Self-made solutions were filtered through a 0.22-μm filter (Millipore, Bedford, MA, USA) and stored at 4 °C or at −20 °C until use. Pipette tips, centrifuge tubes and petri dishes were purchased in aseptic packages and are all disposable.

### Preparation of nuclear transfer donor cells

The preparation of nuclear transfer donor cells were performed as described previously (Liu et al. 2009, 2010, 2014; Zhu et al. 2014, 2016). Briefly, the ear tissue biopsy obtained from an adult Chinese Guike No. 1 breeding boar (earmarked as #200) was washed several times with Dulbecco’s phosphate-buffered saline (DPBS; Gibco, Grand Island, NY, USA) and digested in 0.25 % (w/v) trypsin–EDTA solution for 30 min at 37 °C. Cell suspension was filtered using a 70 µm nylon cell strainer (BD Bioscience, Bedford, MA, USA) and pellets were collected by 1000 rpm centrifugation for 5 min. Cells were seeded onto a 6-well cell culture cluster (NUNC, Shanghai, China) in cell culture medium [Dulbecco’s modified Eagle medium (DMEM; Gibco) supplemented with 15 % (v/v) fetal bovine serum (FBS; Gibco), 100 IU/mL penicillin G and 100 µg/mL streptomycin], then incubated at 37 °C in a Heracell 150i incubator (Thermo Scientific, Waltham, MA, USA) with humidified atmosphere of 5 % (v/v) CO_2_ in air. The fibroblasts were passaged when the primary cells reached a confluence of 80–90 %. Cells were washed twice with DPBS after the medium was discarded, and then 0.5 mL trypsin was added to each well for a 5 min digestion. When most of the cells appeared round or floated off the wall as observed under the microscope, digestion was terminated by adding 2 mL of culture medium. We created a cell suspension by gently pipetting, and then cells were collected by centrifugation at 1000 rpm for 5 min. The supernatant was discarded, and pellets were diluted 1:3 with culture medium. Cells were then mixed well and transferred to 6-well plates. When the cells grew to 80–90 % confluence, the fibroblasts were digested and collected with freezing medium [90 % FBS plus 10 % dimethylsulfoxide (DMSO)]. Finally, the fibroblasts were aliquoted into 2-mL cryogenic tubes (Kirgen, Shanghai, China) and stored in liquid nitrogen for future use.

To prepare nuclear transfer donor cells, cryo-preserved fibroblasts were thawed and cultured for 2 days, following synchronization by serum starvation (DMEM supplemented with 0.5 % FBS) for 48 h. The cells were then harvested and re-suspended with 1 mL micromanipulation medium (10 mM HEPES-buffered TCM-199 containing 0.3 % [w/v] bovine serum albumin [BSA]; pH = 7.3). This cell suspension was maintained at room temperature and used as nuclear transfer donor cells.

### Preparation of nuclear transfer recipient oocytes

In vitro-matured porcine oocytes were used as nuclear transfer recipients and prepared according to methods described previously (Liu et al. 2014; Zhu et al. 2016). Briefly, cumulus-oocyte complexes (COCs) were aspirated from the follicles with sizes of 3–8 mm, and washed twice in PVA-TL-HEPES medium. The COCs were transferred into 200 µL drops of preheated maturation medium (bicarbonate-buffered TCM-199 supplemented with 0.1 % [w/v] polyvinyl alcohol [PVA], 3.05 mM d-glucose, 0.91 mM sodium pyruvate, 0.57 mM cysteine, 10 ng/mL epidermal growth factor [EGF], 0.5 µg/mL follicle-stimulating hormone [FSH], 0.5 µg/mL luteinizing hormone [LH], 0.0750 g/L penicillin G, 0.0500 g/L streptomycin and 10 % [v/v] porcine follicular fluid [PFF]; pH = 7.3), covered with mineral oil, and then incubated for 20–22 h at 38.5 °C in a Forma Series II water jacketed incubator (Thermo Scientific, Marietta, OH, USA) with humidified atmosphere of 5 % (v/v) CO_2_ in air. Then, the COCs were cultured for an additional 20 h in the same medium without the gonadotropins. Following maturation, expanded cumulus cells were removed from the oocytes by vigorous pipetting in the presence of 0.1 % (w/v) hyaluronidase. Oocytes with an evenly granulated ooplasm and an extruded first polar body were selected and placed into the micromanipulation medium drop (containing donor cells and 7.5 µg/mL cytochalasin B) on a 60-mm cell culture dish (NUNC) covered with mineral oil for using as nuclear transfer recipients.

### Construction of cloned porcine embryos

SCNT was performed as described previously (Liu et al. 2014; Zhu et al. 2016). Briefly, maturated oocyte was enucleated under the Nikon Eclipse Ti microscope (Nikon Instruments Inc., Tokyo, Japan) equipped with a Narishige micromanipulator (Narishige Instruments, Tokyo, Japan) by aspirating the first polar body plus a portion of the adjacent cytoplasm (presumably containing the metaphase II plate) using a sharp beveled glass pipette (WPI; Sarasota, Florida, USA) with a diameter of 20–25 µm. After enucleation, a donor cell was injected into the perivitelline space with care taken to maximize the amount of cell membrane contact between the donor cell and the oocyte. The fusion and activation of nuclear-transferred embryos were performed simultaneously using electrical pulses (2 successive DC pulses of 1.2 kV/cm for 30 μs) under an ECM 2001 electro cell manipulator (BTX Inc., San Diego, CA, USA) in a fusion medium (0.3 M Mannitol, 1.0 mM CaCl_2_·2H_2_0, 0.1 mM MgCl_2_·2H_2_0, 0.5 mM HEPES plus 0.3 % [w/v] BSA; pH = 7.3). Fusion was checked at 40–60 min later, and fused embryos were treated with 7.5 µg/mL cytochalasin B for 3 h to suppress extrusion of the pseudosecond polar body. After that, the reconstructed embryos were placed into Porcine Zygote Medium-3 (PZM-3) containing 0.3 % (w/v) BSA and cultured at 38.5 °C in a humidified atmosphere of 5 % (v/v) CO_2_ in air and were cultured for evaluating the full in vitro development.

Cloned embryos were examined for cleavage and blastocyst formation rates on day 2 and 6 (the SCNT were performed on day 0), respectively. The blastocysts were fixed and stained at room temperature in 4 % paraformaldehyde containing 10 µg/mL Hoechst 33342 for 30 min, and then mounted on slides with glycerol. Fluorescent images were captured with a NIS Elements image system (Nikon, Tokyo, Japan) under a Nikon 50i fluorescence microscope (Nikon, Tokyo, Japan), and then processed and analyzed with Photoshop CS5 software (Adobe Systems Inc., San Jose, CA, USA).

### Production of SCNT derived piglets

For generation of cloned pigs, a surgical embryo transfer was performed. Briefly, about two to three hundreds of cloned embryos were cultured 0–1 day in vitro after activation, then surgically transferred into the oviductal ampullary-isthmic junction of the surrogates exhibiting natural estrus (within 1 day of the onset of estrus) using a medical embryo transfer catheter (Weigao group, Weihai, Shandong, China). Pregnancy was diagnosed using a Honda HS-2200V veterinary ultrasound machine (Honda electronics Co., Ltd., Aichi, Japan), and surrogates were delivered by natural parturition on Day 114–120 of gestation (SCNT was performed on Day 0). Newborn piglets were taken care of by a very experienced veterinarian and were weaned at 28 days of age. The birth and weaning weight was measured and compared with contemporary piglets generated by conventional reproduction from the same pig farm.

### DNA parentage analysis

For confirming the piglets were exactly cloned from the donor boar, a DNA microsatellite genotyping analysis was performed as Wei et al. (2013) described previously. Biopsies obtained from such SCNT derived piglets, donor boar and the surrogate sows were collected and processed for isolating of genomic DNA, followed by that, it was sent to the Shanghai GeneCore BioTechnologies Co. Ltd, a widely approved professional organization for animal parentage verification. PCR primers designed for targeting ten porcine-specific microsatellite markers (S0026, S0070, S0155, S0226, SW24, SW72, SW122, SW830, SW857 and SW936) were labeled with the fluorescent dye carboxyfluorescein (FAM), followed by that, used in analysis for determining the genetic relationship among donor boar, SCNT derived piglets as well as embryo transfer surrogate recipients.

### Statistic analysis

Birth and weaning weights of the nuclear transfer derived and control pigs were expressed as mean ± standard deviation (SD) and were analyzed by *t* test using the SPSS18.0 software (SPSS Inc., Chicago, IL, USA). Values of *P* less than 0.05 were considered to be statistically significant.

## Results

### Preparation of fibroblasts from the ear biopsy of an adult Chinese Guike No. 1 breeding boar

**Fig. 1:**
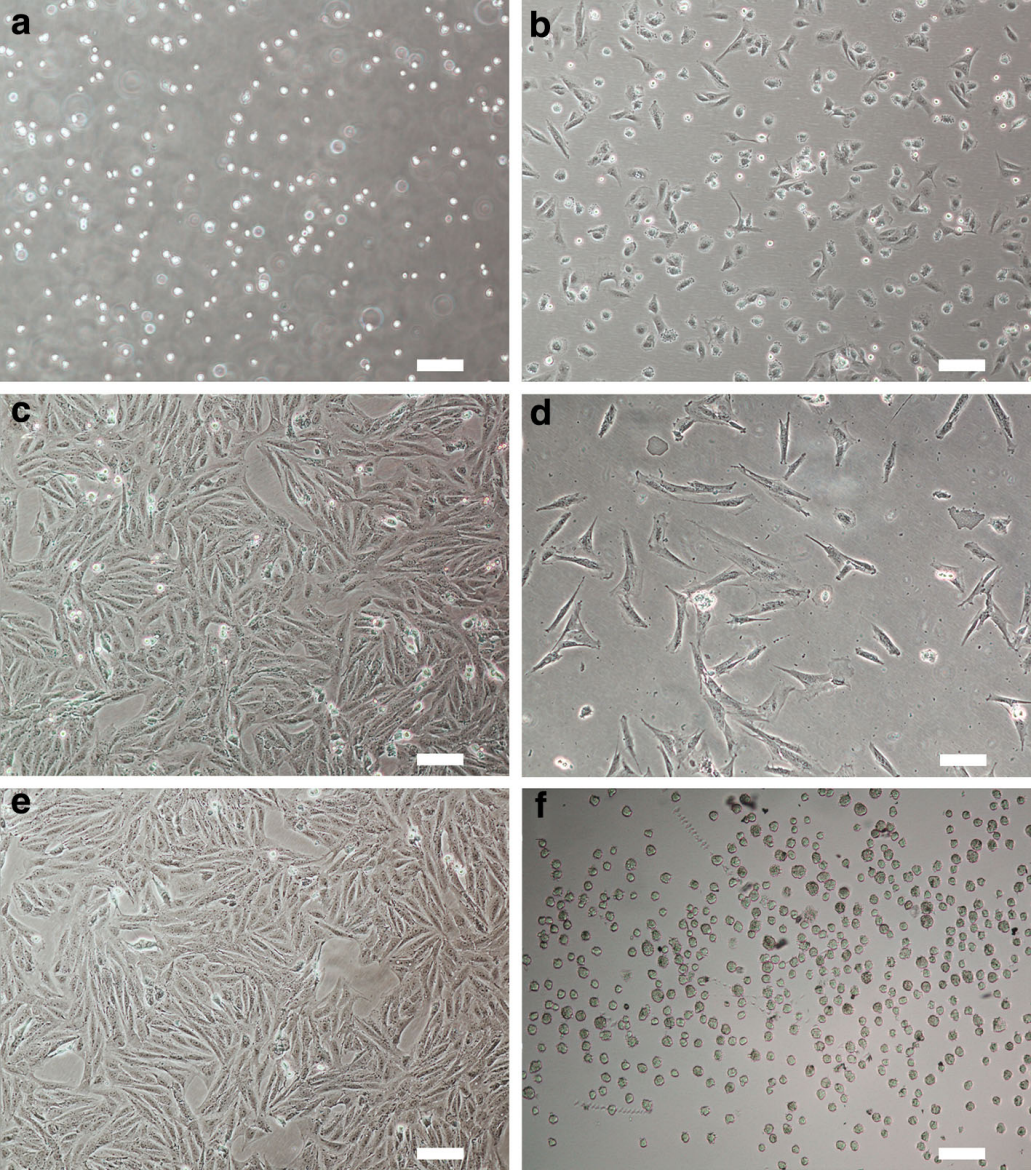
Isolation of somatic fibroblasts from an adult Chinese Guike No. 1 breeding boar. **a** Pellets obtained by trypsin digestion of ear tissue from an adult Chinese Guike No. 1 breeding boar; **b** isolated cells adhered to the culture dish several hours after seeding; **c** primary fibroblasts reached confluence on day 5; **d** cells began to divide and proliferate 1 day after passaging; **e** passaged cells reached confluence and showed a typical fibroblastic morphology; **f** fibroblasts suffered serum starvation cultivation were trypsinizated and used in later nuclear transfer (*Scale bars* 100 μm)

As shown in Fig. 1a, by trypsin digestion, a large number of pellets were collected from the ear tissue, and can be adhered to the culture dish several hours after seeding (Fig. 1b). Generally, the primary cells can grow to confluence on day 5 (Fig. 1c). One day after passaging the primary cells, they began to proliferate (Fig. 1d) and three days later they can reach confluence (Fig. 1e). The rapid proliferation of these somatic cells was reflecting their high activity. In addition, as shown in Fig. 1e, almost all cells showed a typical fibroblastic morphology, such as spindle, diamond or triangle shapes, indicating they were exact highly purified fibroblasts. Overall, the fibroblasts isolated from the adult Chinese Guike No. 1 breeding boar were proliferated actively, and can be used as suitable donor cells (Fig. 1f) for nuclear transfer.

### Construction and in vitro development of cloned porcine embryos

**Fig. 2:**
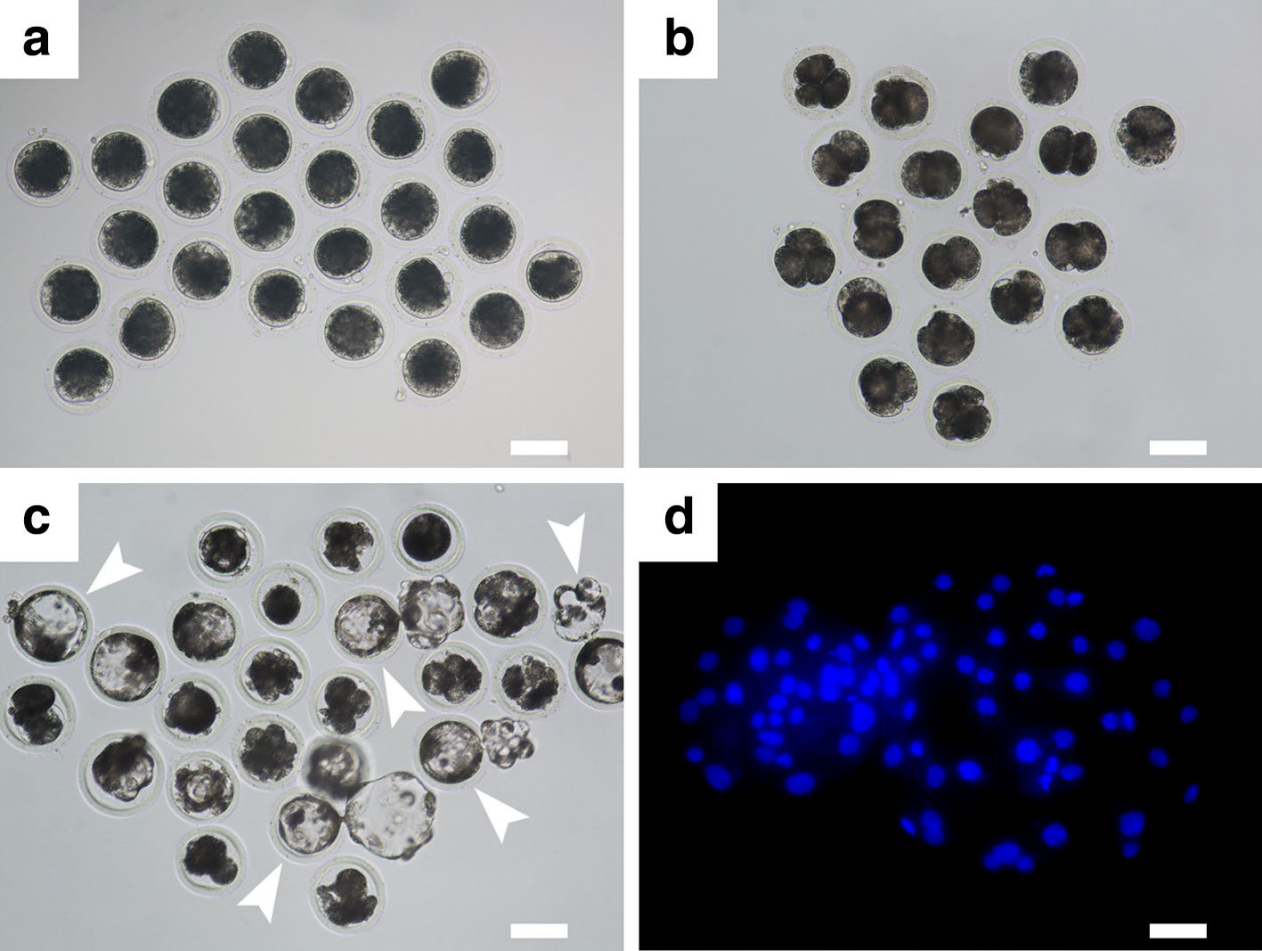
Construction and full in vitro development of cloned porcine embryos. Reconstructed porcine embryos (**a**) derived from the skin fibroblasts of an adult Chinese Guike No. 1 breeding boar can develop to 2–4-cell (**b**) and blastocyst (**c**; indicated by *arrowheads*) stage when cultivated in vitro for 2 and 6 days, respectively. **d** is showing a mounted blastocyst stained with fluorescent dye Hoechst 33342 were detected under a fluorescence microscope with UV excitation for counting the cell number. (*Scale bars* represented as 100 μm in **a**–**c** and 25 μm in **d**, respectively)

As shown in Fig. 2, the cloned embryos generated from the ear fibroblasts of an adult Chinese Guike No. 1 breeding boar showed full development in vitro, namely can developed to blastocyst stage (Fig. 2c). The cleavage rate at 48 h post activation was 71.6 % (144/201, three independent replicates), the blastocyst formation rate at 6 day post activation was 18.4 % (37/201) and the average cell number of blastocysts was 39.0 ± 8.5 (mean ± SD, *n* = 20). The in vitro development efficiency of such cloned embryos was comparable to our previous study aimed at the production of cloned and transgenically cloned embryos from the Huanjiang Xiang pig, which is a unique mini-pig breed originating in south China (Zhu et al. 2016).

### Production of SCNT derived piglets

**Fig. 3:**
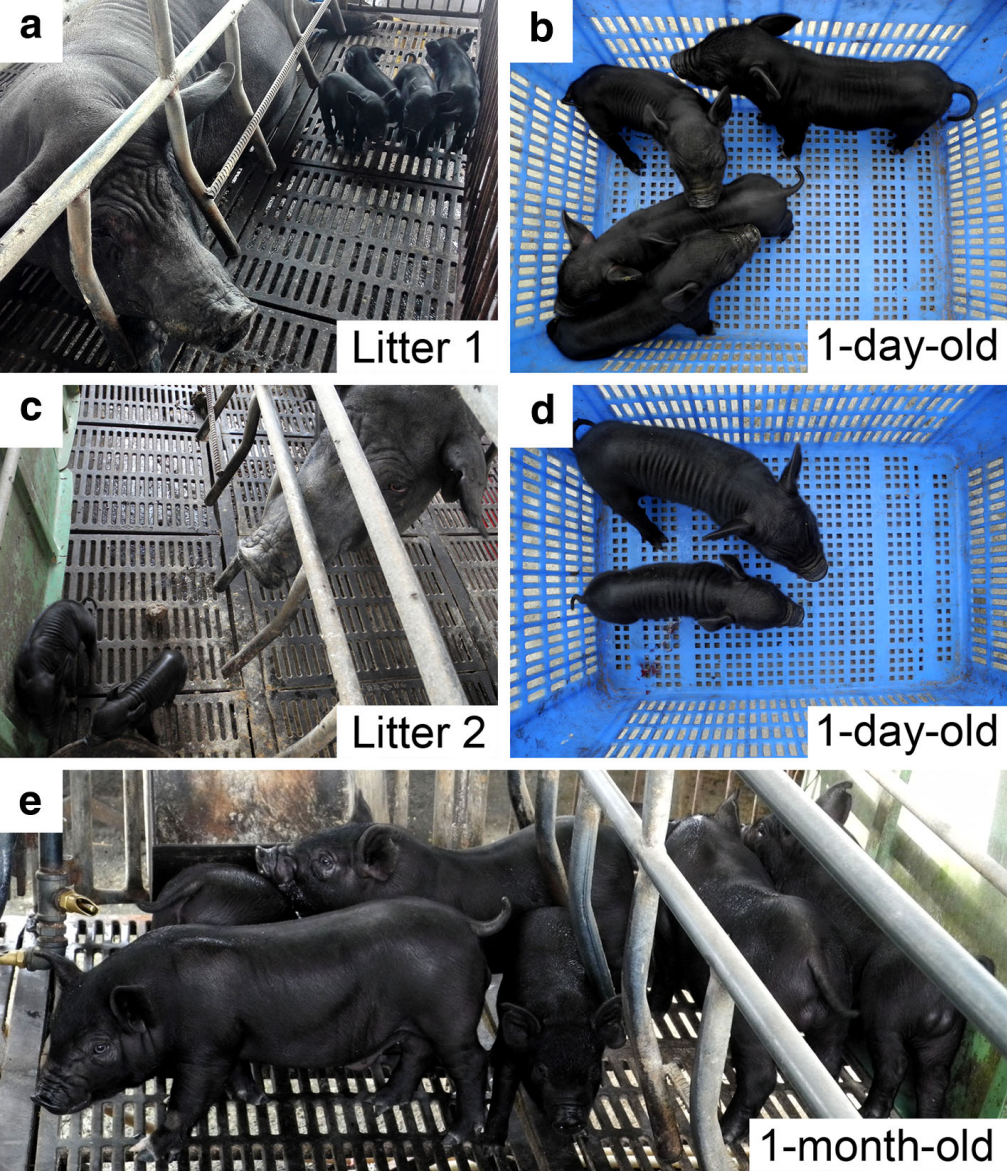
Production of nuclear transfer derived piglets. Two litters of six nuclear transfer derived piglets appeared normal at the birth (**a**, **c**) and the 1 day after birth (**b**, **d**). All of the six SCNT derived piglets were clinically healthy and showed normal development at 1 month of age (**e**)

When 1187 reconstructed embryos in total had been transferred to four surrogate recipients, two of them became pregnant and gave birth to six live male offspring (Table 1). All of the six cloned piglets appeared normal at the birth (Fig. 3a, c) and 1 day after birth (Fig. 3b, d). The overall cloning efficiency was approximately 0.5 % (6/1187). As shown in Table 2, the average birth and weaning weight of six piglets was 1.1 ± 0.4 and 6.8 ± 1.7 kg, respectively. There was no significant difference in birth and weaning weights between the SCNT derived piglets and the contemporary piglets generated by conventional reproduction from the same pig farm (1.1 ± 0.4 vs. 1.2 ± 0.3 and 6.8 ± 1.7 vs. 6.1 ± 0.9, respectively; *P* > 0.05; Table 2). Furthermore, as shown in Fig. 3, all SCNT derived piglets were clinically healthy and developed normally at birth and 1 month of age, suggesting the somatic cloning technique may be a safe and effective tool for rapid expansion of the population of Chinese Guike No. 1 pig breed.

**Table 1:** Production of nuclear transfer derived piglets

Recipient no.	No. of embryos transferred	Day 40 pregnancy status^a^	Gestation period (day)	No. of piglets delivered	Cloning efficiency (%)^b^
#9032	250	+	117	4	1.6
#9849	256	+	118	2	0.8
#0245	328	−	−	−	−
#4574	353	−	−	−	−
Total	1187			6	0.5

^a^Symbols: +, pregnant; −, not pregnant. Pregnancy was determined using a ultrasound scanning at the 40 days after embryo transfer

^b^Cloning efficiency: no. of piglets born/no. of embryos transferred × 100 %

**Table 2:** Comparison of birth and weaning weight of nuclear transfer derived piglets with those of contemporary piglets from conventional breeding

Offspring group	Birth weight (kg)^a^	Weaning weight (kg)^a^
Nuclear transfer	1.1 ± 0.4 (*n* = 6)	6.8 ± 1.7 (*n* = 6)
Conventional breeding	1.2 ± 0.3 (*n* = 64)	6.1 ± 0.9 (*n* = 54)
	*P* = 0.844	*P* = 0.143

^a^Birth and weaning weights were expressed as mean ± standard deviation (SD) and differences between two offspring groups were analyzed by *t* test. Values of *P* less than 0.05 were considered to be statistically significant

### DNA parentage analysis

**Fig. 4:**
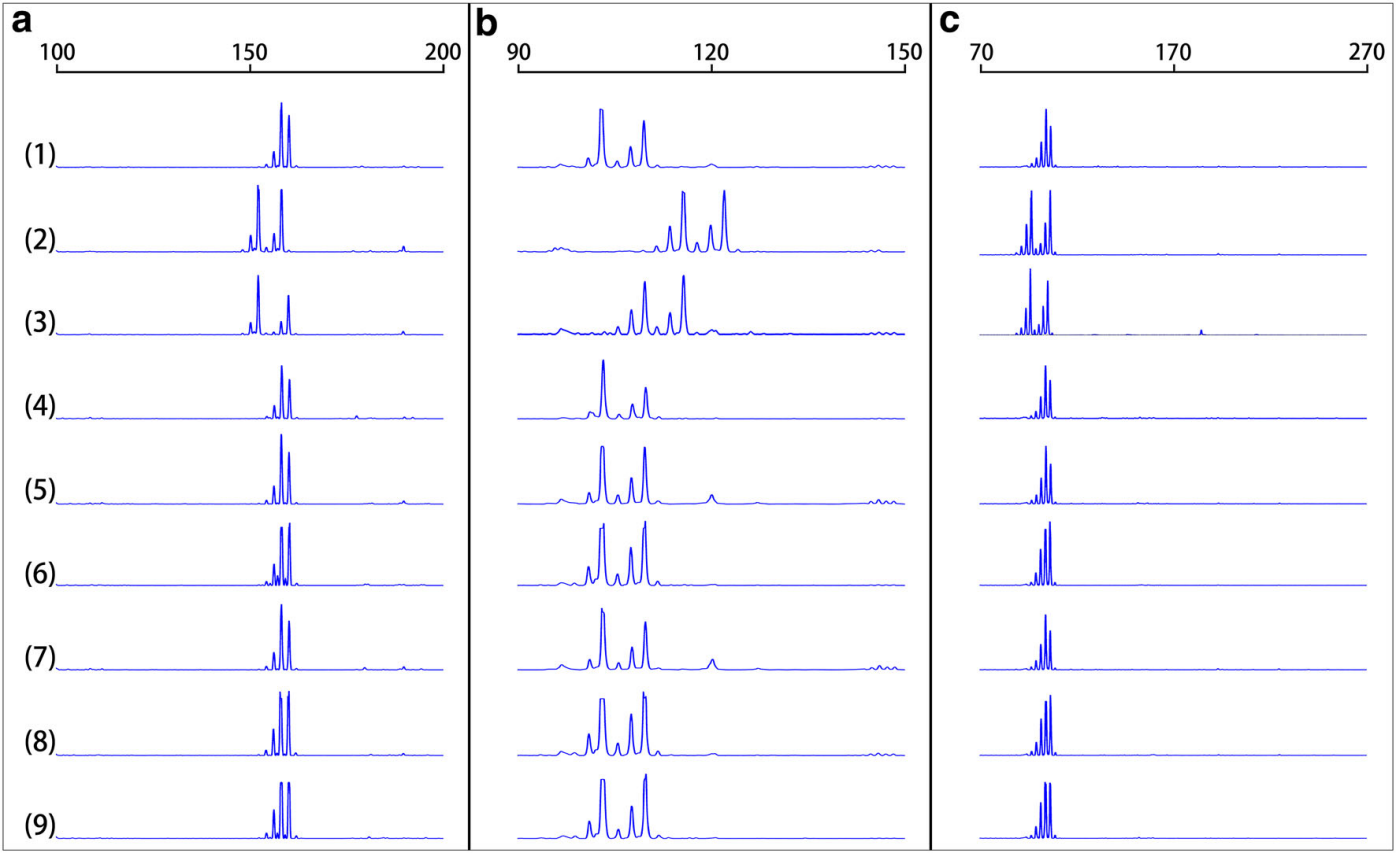
Representative PCR analyses of microsatellite loci. The representative electropherograms represent analyses of three randomly selected microsatellite loci (S00155, SW024 and SW936) in genomic DNA from the donor boar (*1*), surrogate sow #9032 (*2*), surrogate sow #9849 (*3*), as well as the six nuclear transfer derived offspring (*4*–*9*). Each panel shows data for a randomly selected microsatellite-specific primer pair: S00155 (**a**), SW024 (**b**) and SW936 (**c**). Traces were produced on an ABI 3730 DNA analyzer (Applied Biosystems, Foster City, CA, USA). The calculated sizes (in base pairs) are displayed at the top of the traces

DNA microsatellite genotyping analysis was performed on the SCNT derived piglets and their surrogate mothers to confirm identity to the nuclear transfer donor cells isolated from the donor adult Chinese Guike No. 1 breeding boar. As shown in Table 3, by examining with ten porcine-specific microsatellite markers, the genotype of each piglet was identical to the donor cells but different from its surrogate mother. The Fig. 4 was showing the visualized microsatellite genotyping analysis of SCNT derived offspring.

**Table 3:** DNA microsatellite analysis of the nuclear transfer derived piglets

Microsatellite locus	Donor boar (#200)	Surrogate sows		Litter 1 (from #9032)				Litter 2 (from #9849)	
		#9032	#9849	#1000	#1001	#1002	#1003	#1004	#1005
S00026	92/96	93/96	94/96	92/96	92/96	92/96	92/96	92/96	92/96
S00070	277/285	278/283	275/283	277/285	277/285	277/285	277/285	277/285	277/285
S00155	158/160	152/158	152/160	158/160	158/160	158/160	158/160	158/160	158/160
S00226	179/194	178/207	178/192	179/194	179/194	179/194	179/194	179/194	179/194
SW024	102/109	115/121	109/115	102/109	102/109	102/109	102/109	102/109	102/109
SW072	111/115	96/98	110/114	111/115	111/115	111/115	111/115	111/115	111/115
SW122	110	109/111	82/87	110	110	110	110	110	110
SW830	180/182	178/184	179/181	180/182	180/182	180/182	180/182	180/182	180/182
SW857	149/153	148/155	150/155	149/153	149/153	149/153	149/153	149/153	149/153
SW936	107/109	100/110	102/110	107/109	107/109	107/109	107/109	107/109	107/109

For each microsatellite locus, the genotype was determined by size (in base pairs). Two numbers are shown for each sample at each locus represent the PCR product size for each of the two alleles at that particular locus. Litter 1 and 2 were delivered from the surrogate sow #9032 and #9849, respectively

## Discussion

As described above, we successfully produced six healthy cloned male piglets using the ear fibroblasts of an adult breeding boar from the novel Chinese Guike No. 1 swine specialized strain with superior production performance. However, the overall cloning efficiency was only 0.5 %, which was inferior to those in previous studies carried out by other groups (Walker et al. 2002; Zhao et al. 2009, 2010a; Richter et al. 2012; Rim et al. 2013; Huang et al. 2012; Kurome et al. 2013; Callesen et al. 2014; Liu et al. 2015). Such low output of cloned pigs could be largely attributed to the low quality of the transferred cloned embryos generated by reconstruction of enucleated in vitro-matured oocytes with somatic cell nuclei. There is a widespread consensus that the SCNT derived porcine embryos are less ideal than embryos obtained in vivo and even produced via in vitro fertilization (IVF), owing to their lower developmental competence (Vajta and Gjerris 2006; Vajta et al. 2007). For example, as is described above, the blastocyst formation rate of cloned porcine embryos constructed in our laboratory was only 18.4 % (37/201, three independent replicates), while the data from IVF embryos is usually as high as more than 50 % (Yoshioka et al. 2008; Yoshioka 2011; Misumi et al. 2014). Furthermore, in the pig, there is an additional difficulty that at least four good quality embryos are required to initiate and maintain a pregnancy in the surrogate sow (Polge et al. 1966), therefore, the SCNT derived porcine embryos with poor developmental competence may certainly result in a poor outcome of cloned pigs.

Investigations have been addressed that the developmental competences of porcine SCNT-derived embryos are affected by a large number of factors. Among them, an important role seems to be played by the quality of in vitro-matured pig oocytes used as nuclear recipient cells (Dang-Nguyen et al. 2011; Ju and Rui 2012; Pribenszky et al. 2012; Alvarez et al. 2015), different approaches to SCNT procedures (Samiec et al. 2003, 2012; Li et al. 2004; Nánássy et al. 2008; Samiec and Skrzyszowska 2010b, 2012b, 2014a) as well as the systems applied to in vitro embryo culture (Nánássy et al. 2008; Yoshioka et al. 2008; Yoshioka 2011). In addition, evidences also suggest the nuclear donor cell type plays a pivotal role in pig cloning (Hao et al. 2009; Lee et al. 2010; Samiec and Skrzyszowska 2010a; Richter et al. 2012; Fan et al. 2013; Wei et al. 2013; Liu et al. 2014, 2015; Samiec et al. 2015). In this study, we used the ear skin fibroblasts isolated from an adult breeding boar as donor cells for producing cloned embryos. In spite of the successful use of such adult somatic cells for production of cloned pigs (Polejaeva et al. 2000; Richter et al. 2012; Wei et al. 2013; Liu et al. 2015), the overall SCNT efficiency in this mammalian species has been reported to be compromised. For example, when Zhao et al. (2009) tried to clone National Institutes of Health (NIH) miniature pigs with definite swine leukocyte antigen (SLA) using adult ear fibroblast cells as SCNT donor cells, none of the six surrogate recipients established a pregnancy. Moreover, the limited pre- and post-implantation developmental potential of porcine and other mammalian species cloned embryos can be biased to a large extent by the incompatibility in an intergenomic communication between donor cell-inherited nuclear DNA, donor cell-descended mitochondrial DNA (mtDNA) fractions and recipient oocyte-derived mtDNA molecules (Samiec 2005a, b; Yan et al. 2010, 2011; Srirattana et al. 2011). Although the exact mechanisms underlying the epigenetic remodeling and reprogramming of somatic cell nuclei in a cytoplasm of both reconstructed oocytes and descendant blastomeres of resultant cloned embryos are still unclear, increasing lines of evidence suggest that improper or incomplete epigenetic modifications of donor nuclear genome, such as DNA methylation and histone acetylation, are closely associated with the low overall efficiency of pig cloning (Zhao et al. 2010b; Whitworth and Prather 2011; Lee and Prather 2013, 2014). Up to now, several types of epigenetic drugs, such as non-specific DNA methyltransferase inhibitors (e.g., 5-aza-2′-deoxycytidine) and non-specific histone deacetylase inhibitors (e.g., trichostatin A, scriptaid and oxamflatin), have been used for epigenomic transformation of in vitro cultured nuclear donor cells, in vitro maturing nuclear recipient oocytes and activated nuclear-transferred oocytes, resulting in not only significant enhancement of the in vitro developmental capacity of porcine cloned embryos (Himaki et al. 2010; Mao et al. 2012, 2015; Ning et al. 2012; Park et al. 2012; Samiec and Skrzyszowska 2010c, 2011, 2012a, 2014b; Xu et al. 2013; Zhou et al. 2013; Cong et al. 2013; Bohrer et al. 2014; Luo et al. 2014; Liang et al. 2015; Samiec et al. 2015; Whitworth et al. 2015), but also the improvement in the efficiency of generating viable cloned offspring in pigs (Zhao et al. 2009, 2010a). Thus, such successful strategy can be used in our future work for producing cloned pigs with a higher efficiency.

Besides the disappointingly low overall efficiency of pig cloning, the frequent developmental abnormalities exhibited by SCNT-derived offspring have been considered as another major cause which hinders the wide application of such emerging assisted reproductive technology (ART) in pig production and breeding (Vajta et al. 2007). Several cloned piglets have been observed with abnormalities, for instance, lacking an anus and tail (Walker et al. 2002), malformations showed by some important organs (Schmidt et al. 2011; Park et al. 2007, 2010), as well as smaller birth size and lower growth rate (Jiang et al. 2007). As has been demonstrated so far, such cloned pigs with severe developmental abnormalities displayed considerable dysregulation in the expression of both nuclear DNA- and mitochondrial DNA-inherited genes (Jiang et al. 2007; Park et al. 2007, 2010, 2015), which may be attributed to the incorrect somatic cell nuclear reprogramming, the variable epigenetic regulation and the deficiencies in the mitochondrial function occurring in a cytoplasm of recipient oocytes (Tian et al. 2008; Park et al. 2015). In the present study, all of the six cloned piglets showed normal health at birth and 1 month of age. In addition, their birth and weaning weights were comparable to that of controls from conventional breeding, suggesting such cloned Chinese Guike No. 1 piglets have a normal growth. However, the above mentioned dysregulation in gene expression and the real epigenetic status should be further tested for understanding the detail of cloned piglets at molecular level.

In summary, the present study successfully produced six healthy cloned piglets from the ear fibroblasts of an adult Chinese Guike No. 1 breeding boar. The overall cloning efficiency was 0.5 %. All of the cloned piglets were clinically healthy and had a normal weight at birth and weaning. Substantial application of the somatic cloning technique in the production and extension of Chinese Guike No. 1 breeding boars needs to persistently monitor the health and development of the cloned piglets (Liu et al. 2010; Hu et al. 2012). More importantly, the superior production performance seen in their clonal provenance should also be evaluated whether could be displayed when the cloned piglets reached their adulthood (Hu et al. 2012), and such study has already been scheduled into our future research work.
